# Anesthetic Management in Metabolic and Bariatric Surgery Among Anesthesiologists: Survey-Based Study in Poland

**DOI:** 10.3390/jcm15103604

**Published:** 2026-05-08

**Authors:** Eliza Dobruchowska-Kęsikowska, Mateusz Wityk, Natalia Dowgiałło-Gornowicz

**Affiliations:** 1 Department of Anesthesiology and Intensive Care, Municipal Hospital, 10-045 Olsztyn, Poland; dobruchowskaeliza@gmail.com; 2 Department of General and Oncological Surgery, Regional Health Centre, 59-300 Lubin, Poland; mateuszwityk@gmail.com; 3 Department of General, Minimally Invasive and Elderly Surgery, University of Warmia and Mazury in Olsztyn, 10-719 Olsztyn, Poland

**Keywords:** bariatric surgery, anesthesia, airway management, multimodal analgesia, perioperative care, hypertension

## Abstract

**Background/Objectives**: Metabolic and bariatric surgery (MBS) is increasingly performed worldwide and requires specific anesthetic management due to the complex physiological alterations associated with severe obesity. Although several international guidelines provide recommendations for perioperative care in bariatric patients, their implementation in routine clinical practice may vary. This study aimed to report anesthetic practices among Polish anesthesiologists providing anesthesia for bariatric procedures. **Methods**: A cross-sectional survey study was conducted in October 2025 among Polish anesthesiologists. The questionnaire consisted of 13 closed-ended questions addressing demographic characteristics, anesthetic management and blood pressure management, including preoperative thresholds for postponement of elective surgery and intraoperative thresholds for pharmacological treatment of hypotension. The survey was distributed via social media platforms. Participation was anonymous and voluntary. **Results**: A total of 71 anesthesiologists participated in the study. The most commonly used intubation device was the Macintosh laryngoscope (57.7%), while videolaryngoscopy was used by 42.2% of respondents. Positive end-expiratory pressure (PEEP) was routinely applied by most respondents, with 63.4% adjusting its level according to patient body weight. Multimodal analgesia components were commonly used, with paracetamol (95.8%), dexamethasone (91.5%), metamizole (90.1%), and lignocaine (84.5%) being the most frequently administered drugs. Most anesthesiologists reported postponing elective surgery when blood pressure exceeded 180/110 mmHg. More experienced anesthesiologists more often considered lower thresholds for postponement of elective surgery (*p* = 0.006). **Conclusions**: Reported practices among surveyed anesthesiologists for MBS in Poland are generally consistent with international recommendations, particularly regarding the use of PEEP. However, variability remains in airway management strategies and the use of videolaryngoscopy, highlighting the need for continued education and broader implementation of evidence-based perioperative protocols.

## 1. Introduction

Metabolic and bariatric surgery (MBS) is an effective treatment for obesity and obesity-related diseases and has been shown to significantly improve quality of life [1]. The globally increasing number of MBS procedures poses significant challenges in perioperative care, especially for anesthesiologists, due to the complex physiological changes associated with severe obesity [2,3,4].

From the anesthesiology perspective, patients undergoing MBS represent a particularly demanding population. Severe obesity is associated with reduced functional residual capacity, obstructive sleep apnea, increased oxygen consumption, difficult airway management, and altered pharmacokinetics of anesthetic agents [5]. Obesity is also associated with metabolic disturbances, including alterations in insulin sensitivity and vitamin D levels, which may be influenced by lifestyle interventions such as diet and physical activity [6]. These physiological changes significantly influence perioperative management, including induction of anesthesia, airway securing, mechanical ventilation strategies, hemodynamic monitoring, and postoperative analgesia [7]. The increased risk of perioperative complications requires an individualized approach and careful preparation of the anesthesiology team, as emphasized in guidelines issued by the American Society of Anesthesiologists (ASA), Society for Obesity and Bariatric Anaesthesia (SOBA) and the Enhanced Recovery After Surgery Society (ERAS Society) [7,8,9]. However, despite the availability of international recommendations, the implementation of these guidelines in routine clinical practice may vary considerably between countries, institutions, and individual anesthesiology teams. Moreover, there are currently no Polish guidelines specifically addressing anesthesiologic management in MBS. While the Polish expert consensus emphasizes the importance of comprehensive perioperative care and a multidisciplinary team approach, it does not provide detailed anesthesiologic recommendations [10]. This gap, even within existing broader recommendations, suggests a clear need for a systematic evaluation of current anesthesiologic practices in this field.

The aim of this study was to explore anesthesiology practices among Polish anesthesiologists in a survey-based study, particularly regarding airway management, ventilation, multimodal analgesia, and blood pressure control.

## 2. Materials and Methods

This was a cross-sectional survey study conducted among Polish specialists and residents of anesthesiology and intensive care. The study took place from 1 to 31 of October 2025. The survey was specifically designed for this research in accordance with applicable guidelines. It included 13 questions comprising both single-choice and multiple-choice items: 3 demographic questions, 4 focused on anesthetic management, and 6 related to HT. The questionnaire was developed by the authors based on clinical experience and current practice considerations. Prior to distribution, it was reviewed for clarity and relevance. No formal validation of the questionnaire was performed. The full questionnaire is provided as Supplementary Material.

The questionnaire included separate items for patients with and without hypertension (HT), as perioperative blood pressure management may differ between these groups. The survey also distinguished between preoperative thresholds for postponement of elective surgery and intraoperative thresholds for pharmacological blood pressure management.

The survey was distributed via social media platforms (Meta, WhatsApp), including professional groups dedicated to anesthesiologists. Additionally, bariatric surgeons were asked to share the survey with anesthesiologists working in their teams. Participation in the study was anonymous and voluntary, and completing the survey was considered as consent to participate. To minimize duplicate responses, each participant was allowed to submit the questionnaire only once. Due to the nature of social media distribution, the exact number of individuals reached, and the response rate could not be determined.

The study was designed as an exploratory, descriptive survey.

### 2.1. Statistical Analysis

Statistical analysis was planned prior to the study. Due to the sample size and distribution of responses across categories, the statistical analysis was primarily descriptive, and inferential results should be interpreted with caution. A descriptive statistical analysis was conducted. Numbers and percentages were used for categorical variables. The chi-square test or Fisher’s exact test were used to compare categorical variables, depending on expected cell counts. A value *p* < 0.05 was considered statistically significant.

### 2.2. Ethical Considerations

The study was conducted in accordance with the ethical standards of the 1964 Declaration of Helsinki and its subsequent amendments. The study was approved by the Bioethics Committee of University of Warmia and Mazury in Olsztyn (15/2025).

## 3. Results

A total of 71 anesthesiologists participated in the survey. Most respondents were specialists with ≤10 years of experience (42.3%) and worked in university hospitals (31.0%), Table 1. The distribution of weekly case volume was balanced, with approximately one-third of respondents performing fewer than 5, 5–10, or more than 10 anesthetic procedures for MBS per week, Table 1.

### 3.1. Anesthetic Management

The most commonly used intubation device was the Macintosh laryngoscope (57.7%), followed by videolaryngoscopy (42.2%), Table 2. PEEP was routinely applied by most respondents, with 63.4% adjusting its level according to patient body weight, while 32.4% used a standard value of 4–6 cmH_2_O, Table 2.

The most frequently administered drugs included paracetamol (95.8%), dexamethasone (91.5%), metamizole (90.1%), and lignocaine (84.5%), Table 2. Low-dose fentanyl (<0.2 mg) was used by 81.7% of respondents, while higher doses (>0.25 mg) were reported by only 14.1%. Anesthetic management did not differ according to the anesthesiologists’ level of experience, type of hospital, or the number of MBS cases performed per week, Table 2.

### 3.2. Blood Pressure Management

The survey included two distinct aspects of blood pressure management: preoperative thresholds for postponement of elective surgery and intraoperative thresholds for pharmacological blood pressure management.

**Figure 1:**
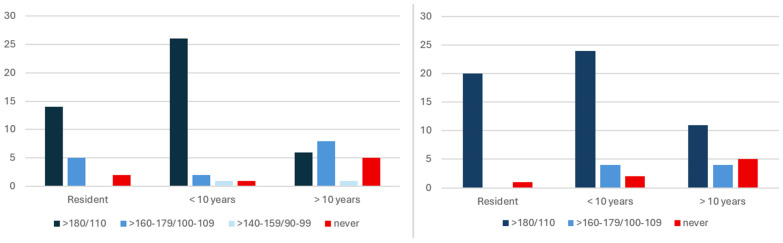
Distribution of preoperative blood pressure thresholds for postponement of elective surgery according to anesthesiologists’ level of experience in patients without HT (**left**) and with HT (**right**). Data are presented as absolute numbers of respondents.

Both in patients with and without pre-existing HT, most anesthesiologists reported postponing elective surgery when blood pressure exceeded 180/110 mmHg (77.5% and 64.8%, respectively), Table 3. However, postponement of elective surgery at lower pressure ranges (160–179/100–109 mmHg) was more frequently observed among patients without HT than those with HT (21.1% vs. 11.3%). The association between the level of experience and thresholds for postponement of elective surgery in patients without and with HT was statistically significant (*p* = 0.006, *p* = 0.030, respectively), Table 3. Less experienced anesthesiologists were more likely to use the 180/110 mmHg threshold almost exclusively, whereas those with >10 years of experience more often considered postponement of elective surgery at lower pressure ranges, Figure 1.

Greater professional experience may be associated with a more individualized and cautious approach to preoperative blood pressure evaluation. No significant differences were found according to hospital type or weekly case volume. In patients without HT, the most commonly reported threshold for ephedrine administration was a 20–30% drop in blood pressure from baseline (47.9%), followed by a decrease of >30% (35.2%), Table 3. In contrast, in patients with HT, the most frequently reported threshold was a 15–20% decrease (38.0%), followed by 20–30% (33.8%).

## 4. Discussion

This study provides an exploratory overview of anesthetic practices reported by Polish anesthesiologists who provide anesthesia for bariatric procedures. To the best of our knowledge, this is the first summary addressing anesthetic management in patients undergoing MBS.

Current Difficult Airway Society (DAS) guidelines recommend videolaryngoscopy as a first-line approach in patients with obesity [11]. Although published after the study period, these guidelines reflect current expert consensus and are used here for contextual interpretation. ASA guidelines for management of difficult airways indicate that videolaryngoscopy provides better visualization of the larynx and a higher first-attempt intubation success rate compared with direct laryngoscopy in patients with predicted difficult intubation [9]. They emphasize the role of videolaryngoscopy as part of the initial, non-invasive airway management strategy aimed at maximizing first-attempt success. In addition, the SOBA guidelines recommend videolaryngoscopy as a first-line technique (Grade A, strong recommendation) [8]. In our study, despite these recommendations, respondents more often declared the use of a classic Macintosh laryngoscope. This may reflect established clinical habits, greater experience with direct laryngoscopy, or limited availability of videolaryngoscopes in some centers. However, this finding should be interpreted with caution, as it is based on self-reported data and does not necessarily reflect actual clinical practice. Additionally, 67% of doctors in training reported using a Macintosh laryngoscope for the first attempt compared with 50% of experienced specialists. However, this difference was not statistically significant (*p* = 0.551).

Appropriate mechanical ventilation is essential in patients with obesity, with PEEP and recruitment maneuvers commonly used to improve gas exchange and lung mechanics [12,13]. Chen et al. demonstrated that PEEP of 10 cmH_2_O may reduce the risk of pulmonary complications [14]. The use of PEEP improved intraoperative oxygenation and respiratory compliance without affecting intraoperative mean arterial pressure (MAP) and was effective in preventing atelectasis. It may have a substantial impact on postoperative outcomes. In our study, 95.8% of respondents used PEEP during anesthesia with 63.4% adjusting its level to the patient’s body weight or BMI, which is consistent with current recommendations. However, the survey did not assess other elements of lung-protective ventilation, such as recruitment maneuvers, tidal volume based on ideal body weight, or ventilation mode.

In the ERAS guidelines, multimodal analgesia is a key component of postoperative pain management, contributing to early mobilization, improved comfort, and faster recovery [7]. For many years, the need for multimodal analgesia both intra- and postoperatively with limited opioid doses has been emphasized. Lidocaine, dexmedetomidine, ketamine, and magnesium, used as part of opioid-free anesthesia, have anti-inflammatory effects compared with classical opioid-based anesthesia and are therefore preferred [15,16,17]. The use of NSAIDs also improves postoperative analgesia and reduces opioid consumption [18,19]. The use of NSAIDs as part of multimodal analgesia may reduce the incidence of postoperative nausea and vomiting (PONV) by decreasing perioperative opioid requirements. In our survey, 81.7% reported using low-opioid analgesia and several non-opioid analgesic agents were frequently reported, including paracetamol, dexamethasone, metamizole, and lignocaine. However, the questionnaire assessed the use of individual drugs rather than a standardized multimodal analgesia protocol. No intraoperative NSAID administration was recorded in the study, mainly due to surgical teams’ concerns about potential bleeding exacerbation and/or the lack of availability of an intravenous formulation. The most commonly used drug was paracetamol, which lacks the adverse effects that limit NSAID use.

One of the questions in our survey concerned the use of lidocaine in the perioperative period. As a component of multimodal analgesia, this drug is recommended for major open and minimally invasive abdominal procedures [7,20]. It is not a universal standard for all operations and requires patient selection, close monitoring, and a consistent safety protocol. Overall, 84.5% of the anesthesiologists surveyed administer lidocaine during anesthesia for bariatric procedures. Limiting factors may include the availability of beds, personnel, and equipment in postoperative care units.

Elective surgery is generally acceptable when BP is ≤160/100 mmHg, whereas surgery should be deferred at BP ≥ 180/110 mmHg (grade 3 HT) or in the presence of hypertensive crisis symptoms or features of cardiovascular instability [21,22]. These recommendations do not apply to emergency procedures. Intraoperatively, the target MAP is usually >65 mmHg. However, in high-risk patients higher MAP values (around 70–75 mmHg) may be beneficial [23]. Even short periods of MAP < 65 mmHg have been associated with an increased risk of acute kidney injury, myocardial infarction, and stroke. Therefore, intraoperative blood pressure should generally be maintained within ±10–20% of baseline values measured after a short rest period in a semi-sitting or ramped position [24].

Hypotension should be treated pharmacologically, most commonly with norepinephrine infusion, phenylephrine boluses in tachycardia, or ephedrine in cases of bradycardia with hypotension. This should be combined with optimization of anesthetic depth, patient positioning, PEEP, and preload. In hypertensive patients, organ ischemia may occur at higher arterial pressure levels, and perioperative blood pressure management should therefore be individualized according to preoperative values [25]. In our study, anesthesiologists administered ephedrine earlier in hypertensive patients, typically at a 15–20% decrease in MAP from baseline.

These findings may suggest potential differences between guideline recommendations and reported clinical practice. The use of appropriate airway management strategies, effective multimodal analgesia, and careful monitoring of vital parameters may improve surgical outcomes, patient safety, and perioperative comfort. An interdisciplinary approach and continuous education of the anesthesiology team remain essential. Future studies with larger cohorts are needed to better evaluate the implementation of guidelines and their potential impact on perioperative outcomes in MBS.

This study has several limitations. The relatively small sample size and voluntary participation may limit the generalizability of the findings. Moreover, the survey was distributed via social media, which may introduce selection bias. The study relied on self-reported practices, which may not fully reflect actual clinical behavior. This recruitment strategy may preferentially include younger or more digitally active anesthesiologists. The findings should therefore be interpreted with caution. Another limitation is that the questionnaire was not formally validated, which may affect the reliability of the responses. Regional differences and institutional protocols were not analyzed. Due to the cross-sectional design, the study does not allow assessment of the impact of these practices on clinical outcomes. Therefore, the results should be interpreted as exploratory and may not be fully representative of all anesthetic practice in Poland.

## 5. Conclusions

This survey provides an exploratory overview of reported practices among anesthesiologists involved in MBS. While many reported practices are consistent with contemporary recommendations, including the routine use of PEEP, some discrepancies remain, particularly regarding airway management strategies and the limited use of videolaryngoscopy. These findings highlight the need for continued education and broader implementation of evidence-based perioperative protocols in bariatric anesthesia.

## Figures and Tables

**Table 1:** Characteristics of respondents.

Variable	Number, n (%)
Experience in anesthesiology	
In training	21 (29.6)
≤10 years as specialist	30 (42.2)
>10 years as specialist	20 (28.2)
Type of hospital	
Municipal	17 (23.9)
County/District	12 (16.9)
Voivodeship	18 (25.4)
University/Clinical	22 (31.0)
Private	2 (2.8)
Number of cases per week	
<5	24 (33.8)
5–10	24 (33.8)
>10	23 (32.4)

**Table 2:** Anesthetic management.

Variable	Number, n (%)	Experience vs. Variable *p*-Value	Hospital * vs. Variable *p*-Value	Cases vs. Variable *p*-Value
Method of intubation		0.551	0.472	0.424
Macintosh laryngoscope	41 (57.7)			
Videolaryngoscope	30 (42.2)			
The use of positive end-expiratory pressure (PEEP) during anesthesia		0.050	0.539	0.435
No	3 (4.2)			
Yes, standard 4–6 cmH_2_O	23 (32.4)			
Yes, adjusted to body weight	45 (63.4)			
Use of drugs:				
Paracetamol	68 (95.8)			
Dexamethasone	65 (91.5)			
Metamizole	64 (90.1)			
Lignocaine	60 (84.5)			
Fentanyl < 0.2 mg	58 (81.7)			
Ondansetron	52 (73.2)			
Ketamine	39 (54.9)			
Oxycodone	31 (43.7)			
Fentanyl > 0.25 mg	10 (14.1)			
Tranexamic acid	8 (11.3)			

* private hospital was excluded from the analysis.

**Table 3:** Blood pressure management.

Variable	Number, n (%)	Experience vs. Variable *p*-Value	Hospital * vs. Variable *p*-Value	Cases vs. Variable *p*-Value
Postponement of elective surgery in patients WITHOUT HT:		0.006	0.206	0.091
140–159 and/or 90–99 mmHg	2 (2.8)			
160–179 and/or 100–109 mmHg	15 (21.1)			
≥180 and/or ≥110 mmHg	46 (64.8)			
Isolated hypertension is not a reason for postponement of elective surgery	8 (11.3)			
Postponement of elective surgery in patients WITH HT:		0.030	0.248	0.064
140–159 and/or 90–99 mmHg	0 (0.0)			
160–179 and/or 100–109 mmHg	8 (11.3)			
≥180 and/or ≥110 mmHg	55 (77.5)			
Isolated hypertension is not a reason for postponement of elective surgery	8 (11.3)			
Ephedrine administration in patients WITHOUT HT:		0.316	0.067	0.818
Drop of 15–20% of baseline value	8 (11.3)			
20–30%	34 (47.9)			
>30%	25 (35.2)			
I do not administer ephedrine	4 (5.6)			
Ephedrine administration in patients WITH HT:		0.387	0.474	0.582
Drop of 15–20% of baseline value	27 (38.0)			
20–30%	34 (33.8)			
>30%	17 (23.9)			
I do not administer ephedrine	3 (4.2)			

* private hospital was excluded from the analysis.

## Data Availability

As the survey was fully anonymous, all data are anonymized. The dataset can be shared by the authors upon reasonable request.

## Supplementary Materials

The following supporting information can be downloaded at: https://www.mdpi.com/article/10.3390/jcm15103604/s1, Anesthesia for Bariatric Surgery—Survey Questionnaire.

## References

[1] Wysocki M., Mizera M., Karpińska I., Ptaszkiewicz K., Małczak P., Pisarska-Adamczyk M., Kania M., Major P. (2024). Analysis of quality of life in patients with clinically severe obesity and type 2 diabetes mellitus after laparoscopic sleeve gastrectomy—A 12-month prospective observational study. Pol. J. Surg..

[2] Ortiz V.E., Kwo J. (2015). Obesity: Physiologic changes and implications for preoperative management. BMC Anesthesiol..

[3] Angrisani L., Santonicola A., Iovino P., Palma R., Kow L., Prager G., Ramos A., Shikora S. (2024). Collaborative Study Group for the IFSO Worldwide Survey. IFSO Worldwide Survey 2020–2021: Current Trends for Bariatric and Metabolic Procedures. Obes. Surg..

[4] Janik M.R., Sroczyński P., Major P. (2024). Bariatric surgery in Poland, 2023: Growth, trends, and impact of the KOS BAR program. Wideochir Inne Tech. Maloinwazyjne.

[5] Hardt K., Wappler F. (2023). Anesthesia for Morbidly Obese Patients. Dtsch. Arztebl. Int..

[6] Alioto A., Rossi C., Capano S., Amato A., Baldassano S., Pagliaro A., Lauriello G., Kuliś S., Proia P. (2023). Biochemical assessment of insulin and vitamin D levels in obese adolescents after diet and physical activity: A retrospective observational study. Biomed. Hum. Kinet..

[7] Stenberg E., Dos Reis Falcão L.F., O’Kane M., Liem R., Pournaras D.J., Salminen P., Urman R.D., Wadhwa A., Gustafsson U.O., Thorell A. (2022). Guidelines for Perioperative Care in Bariatric Surgery: Enhanced Recovery After Surgery (ERAS) Society Recommendations: A 2021 Update. World J. Surg..

[8] McKechnie A., Iliff H.A., Black R., Ahmad I., Chesworth A., Chesworth P., Davis N., Griffiths C. (2025). Airway management in patients living with obesity: Best practice recommendations from the Society for Obesity and Bariatric Anaesthesia: Endorsed by the All Wales Airway Group, Scottish Airway Group and Difficult Airway Society. Anaesthesia.

[9] Apfelbaum J.L., Hagberg C.A., Connis R.T., Abdelmalak B.B., Agarkar M., Dutton R.P., Fiadjoe J.E., Greif R., Klock P.A., Mercier D. (2022). 2022 American Society of Anesthesiologists Practice Guidelines for Management of the Difficult Airway. Anesthesiology.

[10] Major P., Orłowski M., Małczak P., Dowgiałło-Gornowicz N., Binda A., Bogdański P., Budzyński A., Budzyńska D., Janik M., Jaworski P. (2025). Polish Expert Consensus on Metabolic and Bariatric Surgery: 2025 update. Wideochirurgia Inne Tech. Maloinwazyjne.

[11] Ahmad I., El-Boghdadly K., Iliff H., Dua G., Higgs A., Huntington M., Mir F., Nouraei S.R., O’SUllivan E.P., Patel A. (2026). Difficult Airway Society 2025 guidelines for management of unanticipated difficult tracheal intubation in adults. Br. J. Anaesth..

[12] Aldenkortt M., Lysakowski C., Elia N., Brochard L., Tramèr M.R. (2012). Ventilation strategies in obese patients undergoing surgery: A quantitative systematic review and meta-analysis. Br. J. Anaesth..

[13] Costa Souza G.M., Santos G.M., Zimpel S.A., Melnik T. (2020). Intraoperative ventilation strategies for obese patients undergoing bariatric surgery: Systematic review and meta-analysis. BMC Anesthesiol..

[14] Chen C., Shang P., Yao Y. (2024). Evidence in Cardiovascular Anesthesia (EICA) Group. Positive end-expiratory pressure and postoperative pulmonary complications in laparoscopic bariatric surgery: Systematic review and meta-analysis. BMC Anesthesiol..

[15] Brown E.N., Pavone K.J., Naranjo M. (2018). Multimodal General Anesthesia: Theory and Practice. Anesth. Analg..

[16] Mulier J. (2017). Opioid free general anesthesia: A paradigm shift?. Rev. Esp. Anestesiol. Reanim..

[17] Mulier J., Dillemans B. (2018). A Prospective Randomized Controlled Trial Comparing a Multitarget OpioidFree Anaesthesia (OFA) and a 3-Liter Volume Calculated Airseal CarbonDioxide Insufflator with a Balanced Anaesthesia Using Sufentanil-Sevofluraneand a Standard 15 MmHg Carbon Dioxide Pressure PneumoperitoneumInsufflator in a 2x2 Factorial Design. J. Clin. Anesth. Pain. Med..

[18] Govindarajan R., Ghosh B., Sathyamoorthy M.K., Kodali N.S., Raza A., Aronsohn J., Rajpal S., Ramaswamy C., Abadir A. (2005). Efficacy of ketorolac in lieu of narcotics in the operative management of laparoscopic surgery for morbid obesity. Surg. Obes. Relat. Dis..

[19] Beloeil H., Albaladejo P., Sion A., Durand M., Martinez V., Lasocki S., Futier E., Verzili D., Minville V., Fessenmeyer C. (2019). OCTOPUS group. Multicentre, prospective, double-blind, randomised controlled clinical trial comparing different non-opioid analgesic combinations with morphine for postoperative analgesia: The OCTOPUS study. Br. J. Anaesth..

[20] Foo I., Macfarlane A.J.R., Srivastava D., Bhaskar A., Barker H., Knaggs R., Eipe N., Smith A.F. (2021). The use of intravenous lidocaine for postoperative pain and recovery: International consensus statement on efficacy and safety. Anaesthesia.

[21] Gencer B., Gale C.P., Aktaa S., Halvorsen S., Beska B., Abdelhamid M., Mueller C., Tutarel O., McGreavy P., Schirmer H. (2023). European Society of Cardiology quality indicators for the cardiovascular pre-operative assessment and management of patients considered for non-cardiac surgery. Developed in collaboration with the European Society of Anaesthesiology and Intensive Care. Eur. Heart J. Qual. Care Clin. Outcomes.

[22] Hartle A., McCormack T., Carlisle J., Anderson S., Pichel A., Beckett N., Woodcock T., Heagerty A. (2016). The measurement of adult blood pressure and management of hypertension before elective surgery: Joint Guidelines from the Association of Anaesthetists of Great Britain and Ireland and the British Hypertension Society. Anaesthesia.

[23] Zhang K., Liu C., Wang M., Zhang T., Meng B., Yao S., Lou J., Fu Q., Liu Y., Cao J. (2025). Intraoperative Hypotension and Major Adverse Cardiac Events Among Older Adult Patients Undergoing Noncardiac Surgery: Retrospective Cohort Study. JMIR Aging.

[24] Saugel B., Fletcher N., Gan T.J., Grocott M.P., Myles P.S., Sessler D.I., Auzinger G., Chappell D., Edwards M., Forni L.G. (2024). PeriOperative Quality Initiative XI (POQI XI) Workgroup Members. PeriOperative Quality Initiative (POQI) international consensus statement on perioperative arterial pressure management. Br. J. Anaesth..

[25] Futier E., Lefrant J.Y., Guinot P.G., Godet T., Lorne E., Cuvillon P., Bertran S., Leone M., Pastene B., Piriou V. (2017). INPRESS Study Group. Effect of Individualized vs Standard Blood Pressure Management Strategies on Postoperative Organ Dysfunction Among High-Risk Patients Undergoing Major Surgery: A Randomized Clinical Trial. JAMA.

