# Supplementary material for: Anesthetic Management in Metabolic and Bariatric Surgery Among Anesthesiologists: Survey-Based Study in Poland
Source: J Clin Med. 2026 May 8;15(10):3604. doi: 10.3390/jcm15103604 (PMC13207532; doi:10.3390/jcm15103604)
Supplement: Supplementary file 1 [file jcm-15-03604-s001.zip › jcm-4278186-supplementary.pdf]

Anesthesia for Bariatric Surgery – Survey Questionnaire

1. Professional experience (single choice)

a) In training (resident in anesthesiology and intensive care)

b) ≤10 years as a specialist in anesthesiology and intensive care

c) >10 years as a specialist in anesthesiology and intensive care

2. Workplace (single choice)

a) Municipal hospital

b) District hospital

c) Regional (voivodeship) hospital

d) University/teaching hospital

e) Private hospital

3. Average weekly number of bariatric anesthesia procedures in your hospital (single choice)

a) 0–4

b) 5–10

c) ≥11

4. Preferred method for tracheal intubation (single choice)

a) Macintosh laryngoscope

b) Macintosh laryngoscope + introducer (short stylet or bougie)

c) Videolaryngoscope

d) Videolaryngoscope + introducer (short stylet or bougie)

5. Do you apply positive end-expiratory pressure (PEEP) during anesthesia? (single choice)

a) No

b) Yes, routinely 4–6 cmH_2O

c) Yes, adjusted to patient body weight/BMI

6. Please select all medications you typically administer during anesthesia (or as premedication) (multiple choice)

a) Fentanyl 0.1–0.2 mg

b) Fentanyl ≥0.25 mg

c) Oxycodone

d) Paracetamol

e) Metamizole

f) Dexamethasone (Dexaven)

g) Lidocaine

h) Ketamine

i) Magnesium sulfate

j) Clonidine

k) Dexmedetomidine

l) Ondansetron

m) Tranexamic acid (Exacyl)

n) Other: ______

7. If you administer lidocaine during anesthesia: (single choice)

a) As a bolus / rapid infusion

b) As a bolus + continuous IV infusion during the procedure

c) As a bolus + continuous IV infusion during and after the procedure

d) I do not routinely use lidocaine

8. When do you most frequently observe a DROP in blood pressure (>20% from baseline)? (single choice)

a) Intubation

b) Positioning + pneumoperitoneum

c) During the procedure

d) Desufflation

e) Other: ______

9. When do you most frequently observe an INCREASE in blood pressure (>20% from baseline)? (single choice)

a) Intubation

b) Positioning + pneumoperitoneum

c) Desufflation

d) During the procedure

e) Other: ______

10. At what blood pressure values (in patients WITHOUT diagnosed hypertension) would you postpone anesthesia? (single choice)

a) 140–159 and/or 90–99 mmHg

b) 160–179 and/or 100–109 mmHg

c) ≥180 and/or ≥110 mmHg

d) Isolated hypertension is not a reason to postpone

11. At what blood pressure values (in patients WITH diagnosed and treated hypertension) would you postpone anesthesia? (single choice)

a) 140–159 and/or 90–99 mmHg

b) 160–179 and/or 100–109 mmHg

c) ≥180 and/or ≥110 mmHg

d) Isolated hypertension is not a reason to postpone

12. During anesthesia (in a patient WITHOUT a history of hypertension), at what level of blood pressure drop do you administer ephedrine? (single choice)

a) 15–20% from baseline

b) 20–30%

c) >30%

d) I do not administer

13. During anesthesia (in a patient WITH diagnosed hypertension), at what level of blood pressure drop do you administer ephedrine? (single choice)

a) 15–20% from baseline

b) 20–30%

c) >30%

d) I do not administer
